# TMEM176B Prevents and alleviates bleomycin-induced pulmonary fibrosis via inhibiting transforming growth factor β-Smad signaling

**DOI:** 10.1016/j.heliyon.2024.e35444

**Published:** 2024-07-30

**Authors:** Ziwei Wang, Hehua Zhao

**Affiliations:** Department of Pediatrics, Beijing Friendship Hospital, Capital Medical University, Beijing, China

**Keywords:** Pulmonary fibrosis, TMEM176B, TGFβ1

## Abstract

Pulmonary fibrosis is a severe and progressive lung disease characterized by the abnormal accumulation of extracellular matrix, leading to scarring and loss of normal lung function. Recent bioinformatics analysis through the Gene Expression Omnibus (GEO) database identified a significant downregulation of Transmembrane Protein 176B (TMEM176B), previously unexplored in the context of fibrotic lung tissues. To investigate the functional role of TMEM176B, we induced pulmonary fibrosis in mice using bleomycin, TGFβ1, and silica, which consistently resulted in a marked decrease in TMEM176B expression. Intriguingly, overexpression of TMEM176B via adenoviral vectors prior to the induction of fibrosis led to significant improvements in fibrotic manifestations and lung function. Mechanistically, TMEM176B appears to mitigate pulmonary fibrosis by inhibiting the TGFβ1-SMAD signaling pathway, which is a critical mediator of fibroblast proliferation and differentiation and promotes extracellular matrix production. These findings suggest that TMEM176B plays an inhibitory role in the pathophysiological processes of pulmonary fibrosis, highlighting its potential as a therapeutic target.

## 1 Introduction

Pulmonary fibrosis is a chronic, progressive, and fatal interstitial lung disease characterized by irreversible scarring of lung tissue, leading to difficulty in breathing and reduced oxygen supply to the blood [1]. The structural changes associated with pulmonary fibrosis involve the excessive deposition of extracellular matrix (ECM) components, including collagen, leading to the stiffening of the lung tissue and consequent impairment of normal lung function [2]. The global impact of pulmonary fibrosis is substantial, with an estimated prevalence of 13–20 cases per 100,000 individuals for idiopathic pulmonary fibrosis (IPF), the most studied form of the disease [3]. Fibrotic diseases are typically diagnosed through a comprehensive review of the patient's medical history, physical examination, and various tests including imaging tests and lung function tests1. In many cases, a definitive diagnosis requires a biopsy of lung tissue1. Recently, transbronchial cryobiopsy (TBCB) has emerged as a safe and highly effective method for obtaining lung tissue samples in fibrotic diseases [4]. A multicenter study by Freund et al. demonstrated the superiority of TBCB over traditional forceps biopsy in diagnosing both fibrotic and non-fibrotic interstitial lung diseases [5].

The disease progression and prognosis are unpredictable, and most patients survive less than 5 years from the time of diagnosis, underscoring the urgent need for effective therapeutic approaches [6]. The pathogenesis of pulmonary fibrosis is complex and not completely understood. It is thought to be initiated by repetitive injury to the alveolar epithelial cells, followed by abnormal wound healing, fibroblast activation, and ECM deposition [7]. Several environmental, genetic, and age-related factors have been associated with the disease, but the specific mechanisms through which these factors lead to the aberrant fibrotic response remain under investigation [8]. Management of pulmonary fibrosis presents significant challenges. Current therapies, such as antifibrotic drugs, can only slow down the progression of the disease, but not halt or reverse the fibrotic process. Moreover, these treatments are often associated with significant side effects [9]. The ECM, a complex network of proteins and polysaccharides, plays a pivotal role in maintaining lung tissue architecture, and its dysregulation is a hallmark of fibrotic diseases [10]. In pulmonary fibrosis, the ECM not only provides a structural scaffold for the cells but also influences cellular functions such as proliferation, migration, and differentiation [11]. Fibroblasts, the principal cells responsible for ECM production, play a central role in the pathogenesis of pulmonary fibrosis [12]. Under normal conditions, fibroblasts are involved in the maintenance of tissue homeostasis and wound healing [13]. However, in response to injury or stress, these cells may undergo a transformation into myofibroblasts, a process known as activation. These activated myofibroblasts produce excessive amounts of ECM components, contributing to the fibrotic process [14].

Transforming growth factor-beta 1 (TGFβ1) is a potent multifunctional cytokine that is known to play a central role in the pathogenesis of fibrotic diseases including pulmonary fibrosis [15]. The TGFβ1 signaling is initiated when TGFβ1 binds to its receptor, leading to the activation and phosphorylation of receptor-activated SMADs (R-SMADs), SMAD2 and SMAD3 [16]. These R-SMADs then form a complex with SMAD4, which translocates to the nucleus to regulate the transcription of target genes [17]. One of the major downstream effects of the TGFβ1-SMAD signaling pathway includes the regulation of fibroblast proliferation and differentiation into myofibroblasts, a key event in the development and progression of pulmonary fibrosis [18]. Myofibroblasts are known to produce excessive amounts of ECM components, contributing to the fibrotic process [19]. TGFβ1 can also directly stimulate the production of ECM proteins by activating other signaling pathways such as the MAPK pathway [20]. In pulmonary fibrosis, the TGFβ1-SMAD pathway is often overactivated, leading to enhanced fibroblast proliferation, myofibroblast differentiation, and ECM production, which collectively contribute to the fibrotic changes observed in the disease.

TMEM176B (Transmembrane protein 176B) is a tetraspanin membrane protein that belongs to the membrane-spanning 4A (MS4A) protein family [21]. TMEM176B is a poorly characterized protein that has recently emerged as a potential player in immune regulation [22]. Initially identified in dendritic cells, TMEM176B has been associated with the regulation of immune cell activation and inflammatory responses [23]. However, its exact role and the underlying mechanisms remain largely unknown. Recent studies have implicated TMEM176B in various diseases. For instance, in systemic sclerosis, a chronic systemic autoimmune disease characterized by fibrosis in skin and internal organs, TMEM176B was found to be upregulated in fibroblasts and was associated with increased ECM production [24].

Given the existing data, there is a significant gap in our understanding of the role of TMEM176B in pulmonary fibrosis. It is this gap that our study seeks to address, specifically investigating the role and impact of TMEM176B on the TGFβ1-SMAD signaling pathway in the context of pulmonary fibrosis. Our research could potentially shed light on novel therapeutic targets for this severe and progressive lung disease.

## 2 Methods

### 2.1 Human sample

The procurement of human lung tissues was conducted in strict accordance with the ethical standards of the Beijing Friendship Hospital, Capital Medical University's Review Board (Ethics Approval Number: IRB2023-21). All participants provided informed consent prior to sample collection, with documentation stored securely in accordance with institutional guidelines.

Inclusion criteria for pulmonary fibrosis patients comprised a definitive diagnosis based on the American Thoracic Society (ATS)/European Respiratory Society (ERS) criteria, including radiological evidence and, where applicable, histopathological confirmation via biopsy [25,26]. Control samples were collected from individuals undergoing surgery for non-cancerous lung conditions, with no history or clinical signs of pulmonary fibrosis or other interstitial lung diseases.

### 2.2 Animals and treatments

Thirty male C57BL/6 mice, aged 6–8 weeks with an average weight of 18–22 g, used in this experiment were provided by Hunan Slake Jingda Experimental Animal Co., Ltd. All animals were housed under specific pathogen-free conditions at a temperature of 24 ± 2 °C and a relative humidity of 50 ± 10 %.

To induce pulmonary fibrosis, mice in the BLM group received intratracheal injection of 3 mg/kg of BLM (Selleckchem), dissolved in sterile saline, as recommended in the literature [27]. The administration was performed using a microsprayer (Penn-Century, Philadelphia, PA, USA) under mild anesthesia (isoflurane).

Mice were divided into two main experimental groups: BLM-treated group and BLM + TMEM176B AAV-treated group. The BLM + TMEM176B AAV group received concurrent administration of adeno-associated viral vector (AAV) encoding TMEM176B (GeneCopoeia, Rockville, MD, USA) via intranasal instillation. The titer of the AAV is 1 × 1012 vg/mL. AAV treatment was initiated simultaneously with BLM administration and continued for 14 days.

On day 14 and 21, all mice were euthanized under anesthesia with chloral hydrate and died due to an overdose of the anesthetic agent. Lung tissues were collected for further studies.

### 2.3 Cell culture and treatment

The L929 mouse fibroblast cell line was obtained from the Cell Bank of the Chinese Academy of Sciences. The cells were cultured in advanced Dulbecco's Modified Eagle's Medium (DMEM) supplemented with 10 % fetal bovine serum (Gibco, Grand Island, NY) and incubated at 37 °C in a 5 % CO_2_ atmosphere.

### 2.4 Western blot and antibodies

For the Western blot assay, cells were washed twice with PBS and then lysed using RIPA lysis buffer supplemented with phenylmethylsulfonyl fluoride (PMSF) for total protein extraction. The RIPA lysis buffer was obtained from Beyotime Institute of Biotechnology (Shanghai, China), and PMSF was obtained from Sigma-Aldrich (St. Louis, MO, USA). Total protein from mouse lung tissues was extracted using T-PER Tissue Protein Extraction Reagent from Thermo Scientific. Protein concentrations were determined using the BCA Protein Assay kit from Beyotime Institute of Biotechnology (Shanghai, China). A total of 80 μg of protein extracts were separated by SDS-PAGE and transferred onto polyvinylidene difluoride (PVDF) membranes (ISEQ00010, 0.2 μm, Immobilon). The membranes were then incubated overnight at 4 °C with appropriate primary antibodies, followed by appropriate secondary antibodies. Protein bands were visualized using the ChemiDoc XRS + imaging system from Bio-Rad Laboratories, Inc. Densitometry analyses were performed using Image J software. The primary antibodies used in this study were as follows: anti-TMEM176B (KA&M-BIO, QM11876R, 1:500), anti-Collagen I (Abcam, ab34710, 1:1000), anti-Fibronectin (Abcam, ab268021, 1:1000), anti-α-SMA (Abcam, ab32575, 1:1000), and anti-β-actin (Abcam, ab115777, 1:1000).

### 2.5 Quantitative RT PCR (qPCR)

Total RNA was extracted from collected tissues or cells using the Trizol method, following previously described protocols [28]. The quality and concentration of RNA were assessed using a Nanodrop 2000 spectrophotometer (ND-100, Thermo, Waltham, MA). The detection of all mRNA was performed using the AceQ qPCR SYBR Green Master Mix (Vazyme Biotech Co, Nanjing, China) in the ABI 7900HT Real-Time PCR system (Applied Biosystems). The fold changes in expression levels were calculated using the 2^(-ΔΔCt) method and normalized to β-actin as the endogenous control. The following primers were used.

TMEM176B: sense 5′-CAGTCCGCTCACATCAGCAT-3′, antisense 5′-GCTGCCCATAGTGGATTCTGG-3’.

α-SMA: sense 5′-TGCCTTGGTGTGTGACAATG-3′, antisense 5′-TCACCCACGTAGCTGTCTTT-3’.

Collagen I: sense 5′-GCTCCTCTTAGGGGCCACT-3′, antisense 5′-ATTGGGGACCCTTAGGCCAT-3.

Fibronectin: sense 5′-ATGTGGACCCCTCCTGATAGT-3′, antisense 5′-GCCCAGTGATTTCAGCAAAGG-3’.

β-actin: sense 5′-GCTGTGCTATGTTGCTCTAG-3′, antisense 5′-CGCTCGTTGCCAATAGTG-3′.

### 2.6 Histopathology

A portion of the right lung tissue was fixed in 4 % paraformaldehyde (PFA) for 48 h. Subsequently, the tissue was embedded in paraffin and sectioned into 4-μm slices. To evaluate histopathological changes, the tissue sections were stained with hematoxylin and eosin (HE) following the manufacturer's instructions. Masson's trichrome staining (Nanjing Jiancheng Bioengineering Institute, Nanjing, China) was performed to assess the fibrotic areas in the lung sections. Lung fibrosis was scored by an experienced pathologist, who was blinded to the study, using the scoring system previously described by Ashcroft et al. [29].

### 2.7 Hydroxyproline assay

The hydroxyproline assay was used to determine the collagen content in lung tissue. After 21 days of BLM-induced pulmonary fibrosis, mice were sacrificed, and lung tissue homogenates were prepared. The supernatant was obtained by centrifugation at 3000×*g* at 4 °C for 15 min. The concentration of hydroxyproline in the lung tissue supernatant was measured using the Hyp kit (Jinma, Shanghai, China).

### 2.8 Statistical analysis

All experiments were conducted in triplicates. Statistical analysis was performed using Student's t-test (for comparisons between two groups) or one-way analysis of variance (ANOVA) followed by Tukey's multiple comparisons test (for comparisons among more than two groups). The data were presented as mean ± standard deviation (SD). A *p*-value of less than 0.05 was considered statistically significant.

## 3 Results

### 3.1 Identification and Analysis of TMEM176B as a Potential Modulator in Pulmonary Fibrosis

**Fig. 1:**
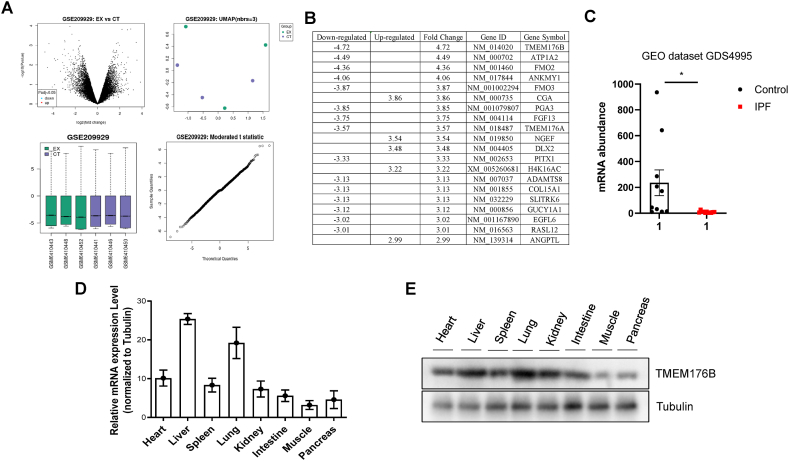
Identification and Analysis of TMEM176B as a Potential Modulator in Pulmonary Fibrosis. (A) A public dataset (GSE209929) was analyzed, and pulmonary fibrosis tissue was compared to adjacent non-pulmonary fibrosis tissues. Volcano plot, UMAP, Boxplot and T-statistic quantile-quantile plot. (B) Heatmap depicting differentially expressed genes in microarray dataset GSE209929. The heatmap illustrates gene expression profiles across various samples, using colors to denote relative expression levels: red for upregulation and blue for downregulation. (C) Comparative analysis of TMEM176B mRNA expression levels between idiopathic pulmonary fibrosis (IPF) samples and normal fibroblast controls, based on GEO dataset GDS4995. (D) Gene expression profile of TMEM176B across various human tissues. qPCR analysis was performed to assess TMEM176B mRNA expression levels in a range of human tissues, with three biological replicates. (E) Protein expression profile of TMEM176B across various human tissues. Western blot analyses were conducted to validate gene expression findings at the protein level, with three biological replicates.

In an effort to identify factors capable of modulating pulmonary fibrosis, we analyzed the GEO database, specifically GSE209929. This data provided lung fibroblasts from PF patients from both non-fibrotic and fibrotic areas, and compared gene expression between these areas by DNA microarray. Data analysis was performed using the GEO2R tools. Volcano plot presents the difference between non-fibrotic and fibrotic tissues after analysis of the datasets with GEO2R. Boxplot and Umap results showed that the upper quartile, median, lower quartile, and distribution of all samples in the normalized are relatively consistent. T-statistic quantile-quantile plot was shown (Fig. 1A). Our analysis of GSE209929 identified a marked downregulation of Transmembrane Protein 176B (TMEM176B) in pulmonary fibrosis (Fig. 1B). To address the concerns raised regarding the reliance on a single dataset for our conclusions on TMEM176B, we sought to validate our findings through the analysis of an independent dataset. Specifically, we compared the mRNA expression levels of TMEM176B in IPF samples to those in normal fibroblasts using the GEO dataset GDS4995. This comparison aimed to confirm the downregulation of TMEM176B in pulmonary fibrosis, as initially observed. The analysis of the GDS4995 dataset revealed a consistent downregulation of TMEM176B mRNA in IPF samples compared to normal fibroblasts, aligning with our original findings from the GSE209929 dataset (Fig. 1C). To further delve into this observation, we performed qPCR analyses to examine the gene expression pattern of TMEM176B across various human tissues. This analysis revealed the highest expression of TMEM176B in lung and liver tissues (Fig. 1C). To validate the gene expression data, we conducted Western blot analyses, which also showed the highest protein expression of TMEM176B in lung, liver, and kidney tissues (Fig. 1D). Taken together, through the analysis of the GEO database, our study identified a significant downregulation of TMEM176B in pulmonary fibrosis and confirmed its highest expression in lung tissues, suggesting a potential role in the pulmonary fibrosis's progression.

### 3.2 Downregulation of TMEM176B and Upregulation of Fibrotic Markers in Pulmonary Fibrosis

**Fig. 2:**
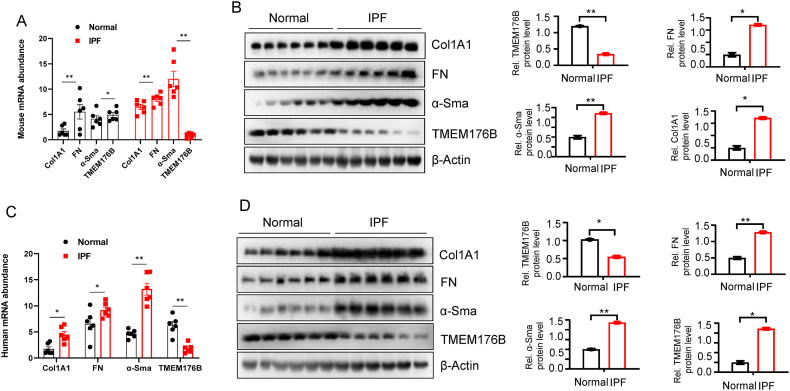
Downregulation of TMEM176B and Upregulation of Fibrotic Markers in Pulmonary Fibrosis. (A) mRNA levels of TMEM176B were measured in pulmonary tissues from healthy individuals and individuals with pulmonary fibrosis. (B) Western blot analysis was utilized to validate the mRNA findings at the protein level. Grayscale analysis results are displayed on the right. (C) A bleomycin-induced mouse model of pulmonary fibrosis was employed. mRNA levels were measured in lung tissues from the control group and fibrotic model. (D) Western blot analysis confirmed the decreased TMEM176B expression at the protein level in lung tissues from the fibrotic model. All data shown in panels A–D are representative of three independent experiments with similar results and expressed as mean ± SEM. Significant differences between groups are indicated by asterisks (*).

In an endeavor to further clarify the expression profile of TMEM176B in human pulmonary fibrosis tissues, we measured the mRNA expression levels of TMEM176B in pulmonary tissues from both healthy individuals and those with pulmonary fibrosis. Our findings revealed a marked decline in mRNA levels of TMEM176B. Concurrently, we observed that expressions of Col1A1 (as a key component of the collagen network), FN (fibronectin, a high-molecular weight glycoprotein of the extracellular matrix), and α-SMA (alpha-smooth muscle actin, a marker for myofibroblast differentiation) were noticeably upregulated in fibrotic lung tissues (Fig. 2A). These findings were further corroborated by Western blot analysis, with the grayscale analysis results shown on the right (Fig. 2B). Additionally, we utilized bleomycin to establish a pulmonary fibrosis mouse model and obtained lung tissue samples from both the control group and fibrotic model. Consistent with our previous findings, both mRNA and protein levels revealed a significant decrease in TMEM176B expression in fibrotic lung tissues (Fig. 2C–D). Our study demonstrates that TMEM176B expression is significantly reduced in human and mouse pulmonary fibrosis, highlighting its potential role in the pathogenesis of the disease.

### 3.3 Efficacy of TMEM176B Overexpression in Reducing Fibrotic Changes Induced by bleomycin and TGFβ1

**Fig. 3:**
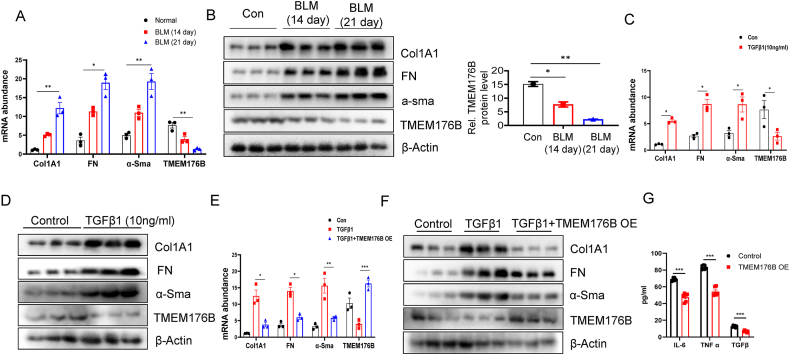
Efficacy of TMEM176B Overexpression in Reducing Fibrotic Changes Induced by Bleomycin and TGFβ1. (A) TMEM176B mRNA levels were quantified in lung tissues of control and Bleomycin-treated mice at day 14 and day 21. (B) Protein expression of TMEM176B was analyzed in lung tissues of control and Bleomycin-treated mice. (C) HLF cells were treated with TGFβ1 and mRNA levels of TMEM176B, Col1A1, FN, and α-SMA were measured. (D) Protein expression levels of TMEM176B, Col1A1, FN, and α-SMA were analyzed in TGFβ1 treated HLF cells. (E–F) HLF cells were treated with TGFβ1 following TMEM176B overexpression. mRNA (E) and protein (F) levels of Col1A1, FN, and α-SMA were significantly suppressed compared to untreated control. (G) Effect of TMEM176B overexpression on inflammatory cytokine secretion in fibroblasts. This figure illustrates the quantification of cytokines IL-6, TNF-α, and TGFβ secreted by fibroblasts following overexpression of TMEM176B. Data are presented as cytokine concentrations in the culture medium, measured using ELISA. Data in A-G are representative of three independent experiments with similar results. The Data are expressed as mean ± SEM, *p < 0.05, **p < 0.01.

Bleomycin is a chemotherapeutic agent, typically used to induce fibrosis [30]. We leveraged this property and administered Bleomycin to C57 mice for 14 and 21 days, respectively. Lung tissue was harvested from both the control group and the Bleomycin-treated groups at day 14 and 21, and analyzed for TMEM176B mRNA levels. We observed a pronounced reduction in TMEM176B mRNA levels in the Bleomycin-treated mice at both time points (Fig. 3A). Parallel findings were noted at the protein level (Fig. 3B). Transforming Growth Factor Beta 1 (TGFβ1), a potent profibrotic cytokine, was utilized to induce fibrotic changes [31]. Upon treatment of HLF cells with 10 ng/ml TGFβ1, we noticed a significant upregulation of fibrosis markers Col1A1, FN, and α-SMA. However, TMEM176B was markedly downregulated (Fig. 3C). The protein expression corroborated with the mRNA findings (Fig. 3D). To determine whether TMEM176B could reverse the fibrotic changes induced by TGFβ1 in HLF cells, we employed an adenovirus to overexpress TMEM176B. Post TGFβ1 treatment, we observed that the mRNA and protein levels of Col1A1, FN, and α-SMA were significantly suppressed, with no noticeable difference from the control group (Fig. 3E–F). To investigate the role of TMEM176B in modulating the inflammatory response of fibroblasts, we conducted experiments aimed at assessing the cytokine profile following TMEM176B overexpression. Our analyses revealed a marked reduction in the secretion of several critical cytokines involved in inflammation, specifically IL-6, TNF-α, and TGFβ (Fig. 3G). These findings suggest that TMEM176B overexpression has a potent anti-inflammatory effect on fibroblasts, potentially contributing to the modulation of inflammatory processes within fibrotic tissues. TMEM176B is markedly downregulated in response to fibrotic stimuli in Bleomycin-treated mice and TGFβ1-treated HLF cells, with overexpression of TMEM176B potentially attenuating fibrosis marker expression, suggesting a protective role against fibrogenesis.

### 3.4 TMEM176B Overexpression Via AAV Attenuates Bleomycin-Induced Pulmonary Fibrosis in Mice

**Fig. 4:**
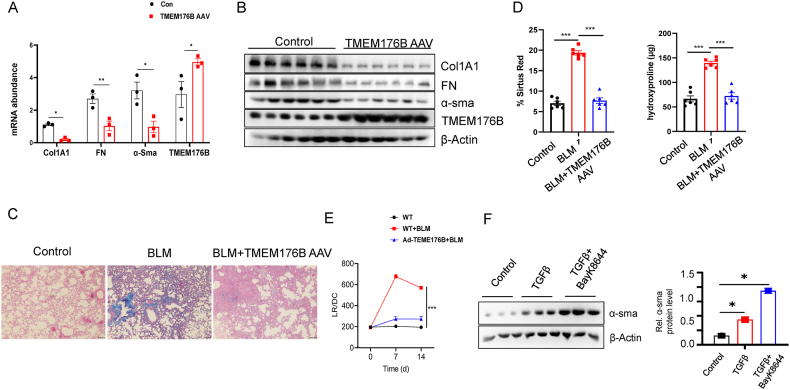
TMEM176B Overexpression Via AAV Attenuates Bleomycin-Induced Pulmonary Fibrosis in Mice. (A–B) Pulmonary fibrosis was induced in mice using bleomycin for 14 days. The TMEM176B AAV group received concurrent administration of AAV for the same duration as bleomycin treatment. This group demonstrated successful overexpression of TMEM176B and significant attenuation of fibrosis compared to controls. (C) Lung tissues from both groups were evaluated and the TMEM176B AAV group showed a marked reduction in fibrosis. (D) Sirius red staining and hydroxyproline content indicated a significant decrease in fibrotic changes in the TMEM176B AAV group. (E) Lung resistance and dynamic compliance demonstrated an improved lung function in the TMEM176B AAV group. (F) Influence of TMEM176B iinhibition on myofibroblast differentiatin ppost-TGFβ1 treatment. This figure displays the effects of inhibiting TMEM176B using BayK8644 on the differentiation of fibroblasts into myofibroblasts after exposure to TGFβ1. Quantitative analysis of myofibroblast markers, including α-SMA, collagen type I, and fibronectin, is shown. Each group included 6 mice. Data in A-F are representative of three independent experiments with similar results. The Data are expressed as mean ± SEM, *p < 0.05, **p < 0.01, ***p < 0.001.

In our continued investigation of the potential beneficial role of TMEM176B in murine pulmonary fibrosis, we employed an adeno-associated viral vector (AAV) to overexpress TMEM176B (TMEM176B AAV). We induced pulmonary fibrosis in two distinct mouse groups using bleomycin, demonstrating successful overexpression of TMEM176B, which significantly attenuated the degree of fibrosis in the TMEM176B AAV group (Fig. 4A–B). A subsequent evaluation of lung tissues from both groups via Masson's trichrome staining further revealed a marked reduction in fibrosis in the TMEM176B AAV group (Fig. 4C). Sirius red, a commonly used stain for detection and quantification of fibrosis, also indicated a significant decrease in the TMEM176B AAV group's fibrotic changes (Fig. 4D). Hydroxyproline, a major component of the protein collagen, which is often used as a biochemical marker of fibrosis [32], was also found to be significantly less in the TMEM176B AAV group (Fig. 4E). The lung resistance and dynamic compliance (LR/DC), parameters reflecting the mechanical properties of the lung and used to assess lung function, also demonstrated an improved lung function in the TMEM176B AAV group (Fig. 4E). To further explore the functional activity of TMEM176B as a cation channel and its role in cellular differentiation within the fibrotic process, we carried out targeted experiments. We employed BayK8644, a specific inhibitor of TMEM176B, to suppress its activity during these trials. Our results indicate that inhibition of TMEM176B significantly influences the transformation of fibroblasts into myofibroblasts. This finding supports the notion that TMEM176B plays a crucial regulatory role in the fibroblast to myofibroblast transition, a key event in the development of pulmonary fibrosis (Fig. 4F). These findings support the notion that TMEM176B overexpression could ameliorate bleomycin-induced pulmonary fibrosis, potentially by modulating critical fibrosis pathways.

### 3.5 TMEM176B Overexpression Mitigates Silica Dioxide-Induced Fibrosis in HLF cells

**Fig. 5:**
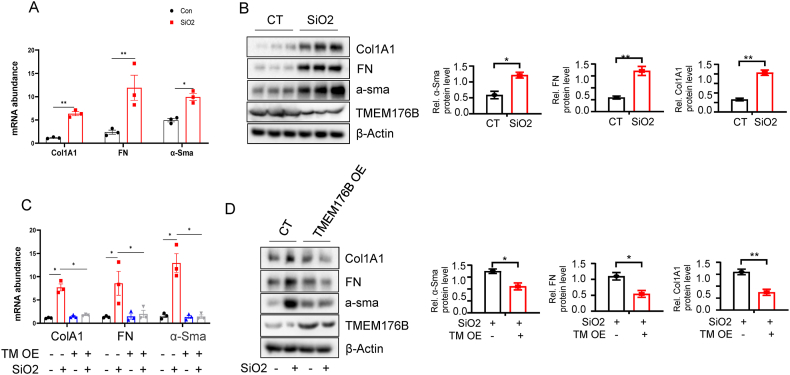
TMEM176B Overexpression Mitigates Silica Dioxide-Induced Fibrosis in HLF Cells. (A) SiO_2_ treatment resulted in a significant upregulation of fibrotic markers at the mRNA level. (B) An increase in fibrotic markers was further confirmed at the protein level in HLF cells treated with SiO_2_. (C–D) Overexpression of TMEM176B in SiO_2_-induced fibrotic cells led to a significant reduction in the severity of fibrosis at both mRNA (C) and protein levels (D). Data in A-D are representative of three independent experiments with similar results. The Data are expressed as mean ± SEM, *p < 0.05, **p < 0.01.

Silica dioxide (SiO2), a commonly employed agent in research, is utilized to induce fibrosis in various cell types [33]. Upon treatment with silica dioxide, we observed a pronounced increase in mRNA levels of fibrotic markers in the HLF cells (Fig. 5A), an upregulation further confirmed at the protein level (Fig. 5B). Remarkably, overexpressing TMEM176B in these silica dioxide-induced fibrotic cells led to a substantial reduction in the severity of fibrosis (Fig. 5C–D). These findings suggest a potential advantageous role of TMEM176B overexpression in silica dioxide-induced fibrosis.

### 3.6 Inhibitory Effect of TMEM176B overexpression on TGF-β-SMAD2/3 Signaling in Pulmonary Fibrosis

**Fig. 6:**
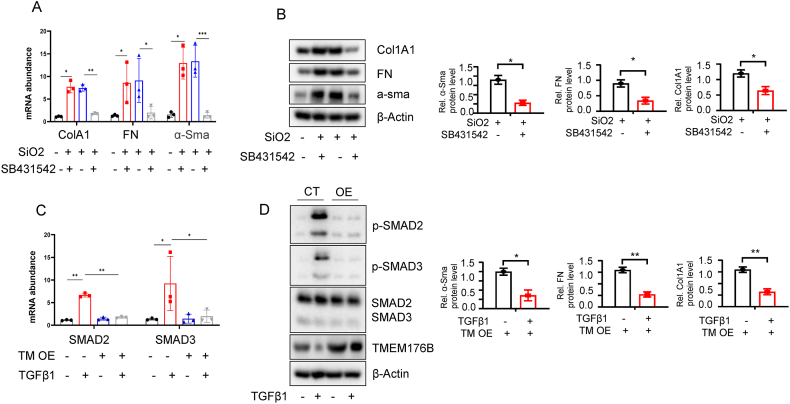
Inhibitory Effect of TMEM176B Overexpression on TGF-β-SMAD2/3 Signaling in Pulmonary Fibrosis. (A–B) Cells co-cultured with SiO2 were treated with SB431542, a specific inhibitor of TGF-β signaling. Post-treatment analysis revealed a significant decrease in the expression levels of fibrotic markers such as α-SMA, collagen I, and Col1A1. (C–D) Fibrotic cells stimulated with TGF-β1 exhibited a marked increase in the phosphorylation of SMAD2 and SMAD3 (*p*-SMAD2 and *p*-SMAD3). Overexpression of TMEM176B in these cells led to a noticeable decrease in the levels of *p*-SMAD2 and *p*-SMAD3. Data in A-D are representative of three independent experiments with similar results. The Data are expressed as mean ± SEM, *p < 0.05, **p < 0.01.

TGF-β-SMAD2/3 signaling is a critical pathway involved in fibrotic processes [34]. To investigate whether the ameliorative effect of TMEM176B on pulmonary fibrosis operates via the TGF-β-SMAD2/3 signaling, we utilized SB431542, an inhibitor of TGF-β. As anticipated, SB431542 reversed the increased expression of α-SMA and collagen I, as well as Col1A1 in co-cultured with SiO_2_-treated Cells (Fig. 6A–B). Moreover, TGFβ1 stimulation resulted in the upregulation of *p*-SMAD2 and *p*-SMAD3 expression. However, following overexpression of TMEM176B and TGFβ1-induced fibrosis, we observed a notable reduction in the levels of *p*-SMAD2 and *p*-SMAD3 (Fig. 6C–D). These findings suggest a potential mechanistic link between TMEM176B overexpression and the mitigation of pulmonary fibrosis through the TGF-β-SMAD2/3 signaling pathway. It also underscores the potential of targeting this pathway for therapeutic interventions in fibrosis-related conditions.

## 4 Discussion

Our study has identified a crucial element in the field of pulmonary fibrosis - a severe and progressive lung disease resulting in scarring and loss of normal lung function. This crucial element, TMEM176B, a transmembrane protein, demonstrated a significant downregulation in fibrotic lung tissues. The revelation, unearthed through diligent bioinformatics analysis using the GEO database, positions TMEM176B as a potential key player in the progression of pulmonary fibrosis. Our observations during the experimental induction of pulmonary fibrosis in mice further reinforced its potential role - we consistently observed a marked decrease in TMEM176B expression. Intriguingly, overexpression of TMEM176B led to noticeable improvements in fibrotic conditions and lung function. Moreover, our mechanistic evaluation indicated that TMEM176B appears to mitigate pulmonary fibrosis by inhibiting the TGFβ1-SMAD signaling pathway.

The role of TMEM176B in lung diseases has been a topic of considerable interest in recent scientific literature. TMEM176B, a transmembrane protein, is known to be involved in the regulation of immune responses by controlling intracellular trafficking in dendritic cells [35]. Previous research has indicated that TMEM176B might play a role in modulating immune response during viral and bacterial infections [36]. In the context of lung diseases, the functional significance of TMEM176B is not fully understood yet. A recent study [37] suggested a potential role for TMEM176B in the pathogenesis of chronic obstructive pulmonary disease (COPD), a common lung condition that shares some common pathological features with pulmonary fibrosis. To date, research specifically linking TMEM176B to pulmonary fibrosis is sparse. Our study is, therefore, a significant contribution to this growing field, providing fresh insights into the role of TMEM176B in the progression of pulmonary fibrosis and setting the stage for future investigations.

The TGFβ-SMAD signaling pathway is a well-known regulator of tissue fibrosis, including pulmonary fibrosis. In a healthy lung, this pathway is tightly regulated and contributes to normal tissue repair and remodeling. However, in pathological conditions such as pulmonary fibrosis, the TGFβ-SMAD pathway becomes overactive, leading to the excessive accumulation of extracellular matrix proteins and fibroblast proliferation, which are hallmarks of the disease [38]. TGFβ, a potent profibrotic cytokine, is found in high levels in fibrotic lungs and is considered a key driver of pulmonary fibrosis. TGFβ signals through its receptors to phosphorylate and activate SMAD proteins, specifically SMAD2 and SMAD3, which then translocate to the nucleus to regulate the transcription of profibrotic genes [39]. The TGFβ-SMAD pathway is not just involved in the initiation of pulmonary fibrosis but is also critical in the progression and maintenance of the fibrotic response [40]. Therefore, understanding the regulation of this pathway and identifying novel regulators, such as TMEM176B as suggested by our study, could provide potential therapeutic targets for this devastating disease.

The mechanism by which TMEM176B mitigates pulmonary fibrosis appears to be related to the inhibition of the TGFβ1-SMAD signaling pathway. Our experimental observations suggest a negative regulatory relationship between TMEM176B and the activation of this pathway, which is a central mediator of fibrotic responses in various tissues, including the lungs. TGFβ1, the most potent profibrotic cytokine, binds to its receptors (TGFβRI and TGFβRII) on the cell surface and triggers the phosphorylation of receptor-activated SMADs (R-SMADs), particularly SMAD2 and SMAD3. These R-SMADs then form a complex with the common SMAD4, translocating to the nucleus to regulate the transcription of numerous genes involved in fibrosis [39]. This cascade leads to the increase in fibroblast proliferation, differentiation into myofibroblasts, and increased production of extracellular matrix (ECM) proteins - all of which contribute to the fibrotic response [40]. In the context of TMEM176B, our study provides preliminary evidence that upregulation of this protein could suppress the TGFβ1-induced SMAD2/3 activation, thereby mitigating the fibrotic process. Overexpression of TMEM176B led to the reduction in collagen production, fibroblast proliferation and differentiation - key features of pulmonary fibrosis. However, the precise molecular interactions between TMEM176B and the TGFβ1-SMAD pathway remain to be elucidated. Our study thus highlights TMEM176B as a potential anti-fibrotic actor, whose role could be harnessed to develop novel therapeutic strategies for pulmonary fibrosis.

Recent studies have indeed suggested a role for TMEM176B in various types of cancer, including lung cancer [3]. For instance, TMEM176B has been shown to inhibit the activation of the NLRP3 inflammasome, linking adaptive and innate anti-tumor responses [4]. This suggests that TMEM176B could play a role in modulating the immune response to tumors, potentially influencing the progression of diseases such as lung cancer. In mouse experimental models, genetic deletion of TMEM176B was associated with controlled tumor progression in EG7 lymphoma, MC38 colon, and LL2 lung cancer [5]. Moreover, anti-tumoral CD8^+^ T cell responses were strongly reinforced in TMEM176B−/− versus WT animals [5]. This indicates that TMEM176B could have a significant impact on the immune response to lung cancer, potentially influencing disease progression and treatment outcomes.

While our study provides valuable insights into the role of TMEM176B in pulmonary fibrosis, there are several limitations to consider. First, the use of animal models, although informative, may not fully replicate the complex human pathophysiology of pulmonary fibrosis, potentially limiting the translatability of our findings. Additionally, the induction of fibrosis was achieved using specific agents such as bleomycin, TGFβ1, and silica, which, while effective, do not encompass all aspects or causes of fibrosis in humans. This could limit the generalizability of our results to other forms or causes of fibrotic disease. Furthermore, our study focused predominantly on the expression and regulation of a single gene, TMEM176B. The interactions of TMEM176B with other pathways and its effects in different cell types within the lung require further exploration to fully understand its role in fibrosis. Finally, although we have shown that overexpression of TMEM176B can mitigate fibrosis in a controlled experimental setting, the efficacy and safety of such an approach in a clinical setting require thorough investigation.

## 5 Conclusions

Our findings have revealed a novel role for TMEM176B in the pathogenesis of pulmonary fibrosis. The study has shown that TMEM176B can inhibit the TGFβ1-SMAD signaling pathway, which is critical for fibroblast proliferation, differentiation, and extracellular matrix production - key features of pulmonary fibrosis. Therefore, TMEM176B may serve as a potential negative regulator of pulmonary fibrosis. These findings have important implications for pulmonary fibrosis treatment. Given the lack of effective therapies for this devastating disease, the identification of TMEM176B as a potential therapeutic target is an exciting development. Modulating the levels or activity of TMEM176B could be a novel strategy to halt or even reverse the progression of pulmonary fibrosis.

## Data availability

All data in this study are true and reliable. The data that support the findings of this study are available from the corresponding author upon reasonable request. The remaining figure data are uploaded as Supplementary information.

## Ethics approval and consent to participate

This study was conducted with approval from the Ethics Committee of Beijing Friendship Hospital of Capital Medical University.

## CRediT authorship contribution statement

**Ziwei Wang:** Formal analysis, Data curation, Conceptualization. **Hehua Zhao:** Writing – review & editing, Writing – original draft, Visualization, Validation.

## Declaration of competing interest

The authors declare no competing interests.
