# Supplementary material for: TMEM176B Prevents and alleviates bleomycin-induced pulmonary fibrosis via inhibiting transforming growth factor β-Smad signaling
Source: Heliyon. 2024 Jul 30;10(15):e35444. doi: 10.1016/j.heliyon.2024.e35444 (PMC11336771; doi:10.1016/j.heliyon.2024.e35444)
Supplement: Multimedia component 1 [file mmc1.pptx]

## Slide 1
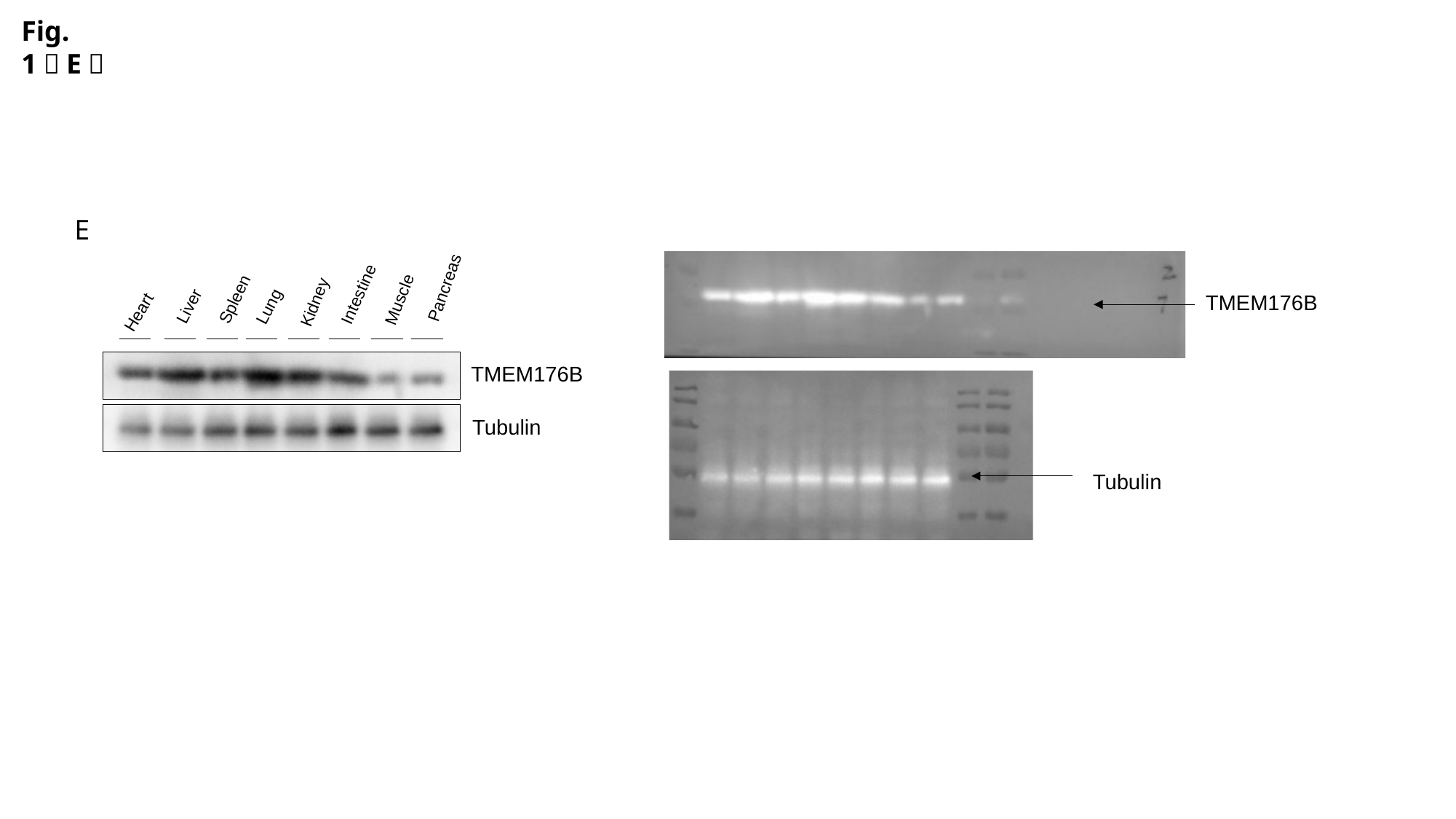

Fig. 1（E）
E
Intestine
Pancreas
Kidney
Spleen
Lung
Muscle
 Heart
Liver
TMEM176B
Tubulin
TMEM176B
Tubulin

## Slide 2
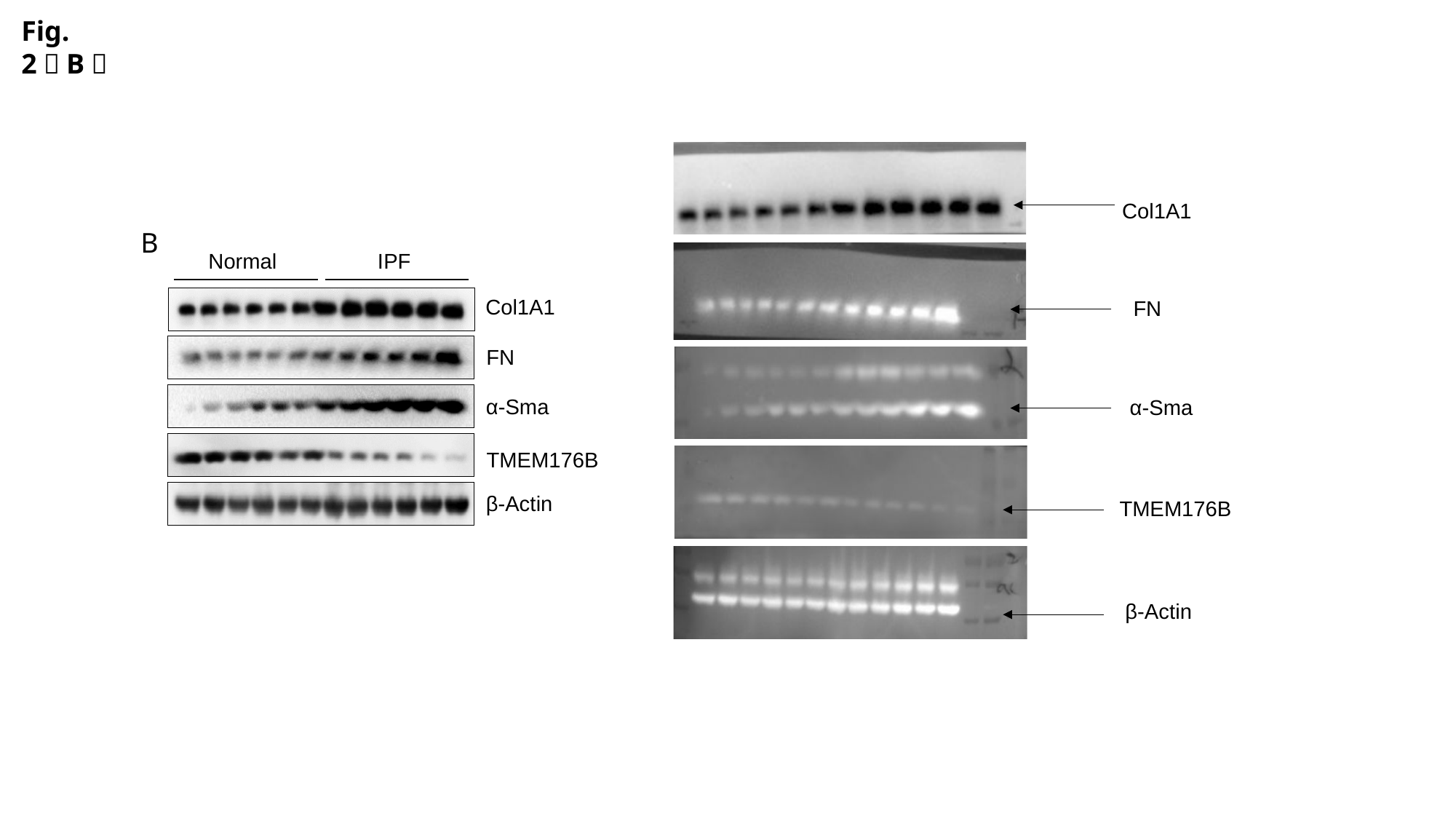

Fig. 2（B）
Col1A1
B
IPF
Normal
Col1A1
FN
α-Sma
TMEM176B
β-Actin
FN
α-Sma
TMEM176B
β-Actin

## Slide 3
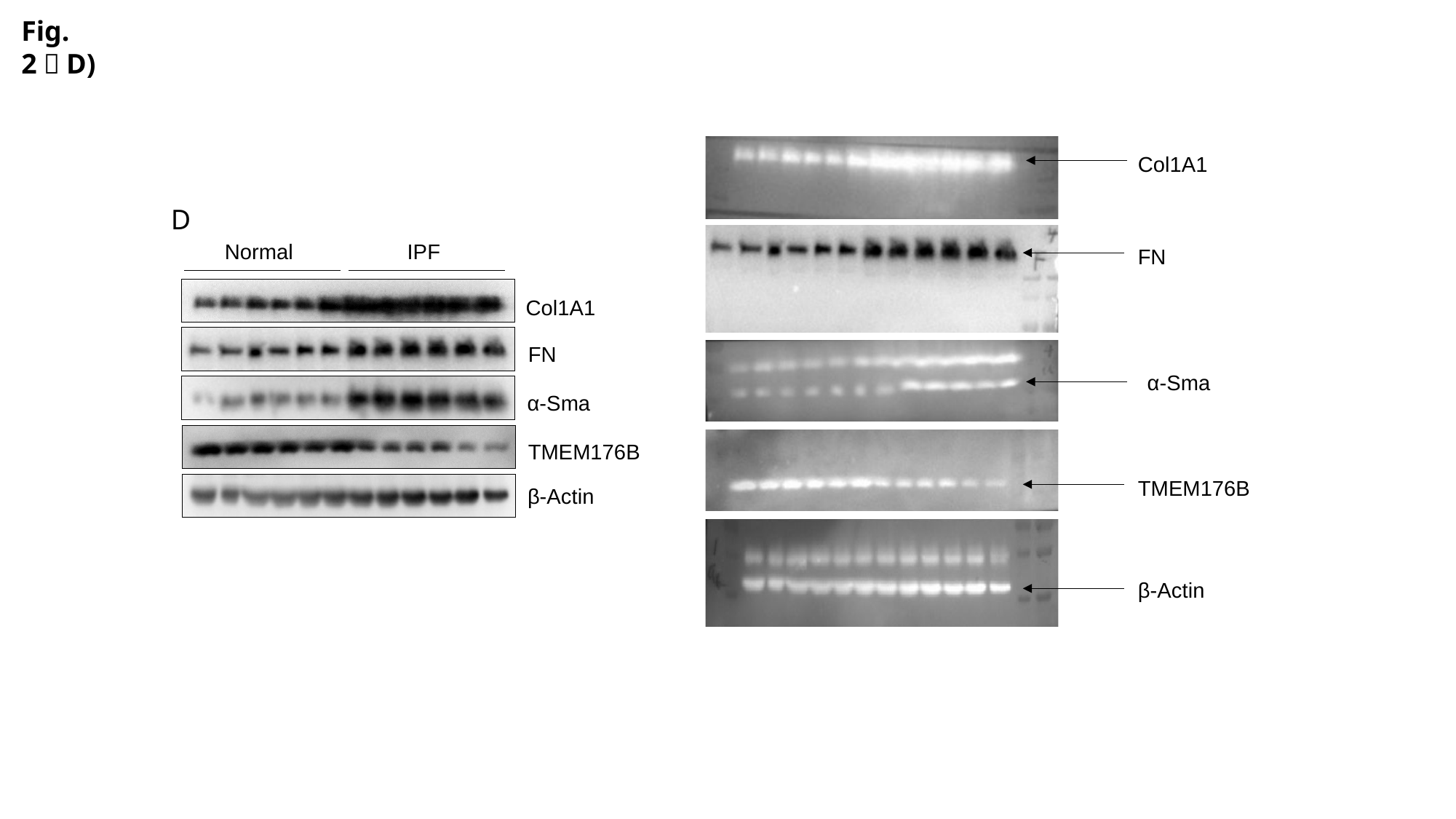

Fig. 2（D)
Col1A1
D
IPF
Normal
Col1A1
FN
α-Sma
TMEM176B
β-Actin
FN
α-Sma
TMEM176B
β-Actin

## Slide 4
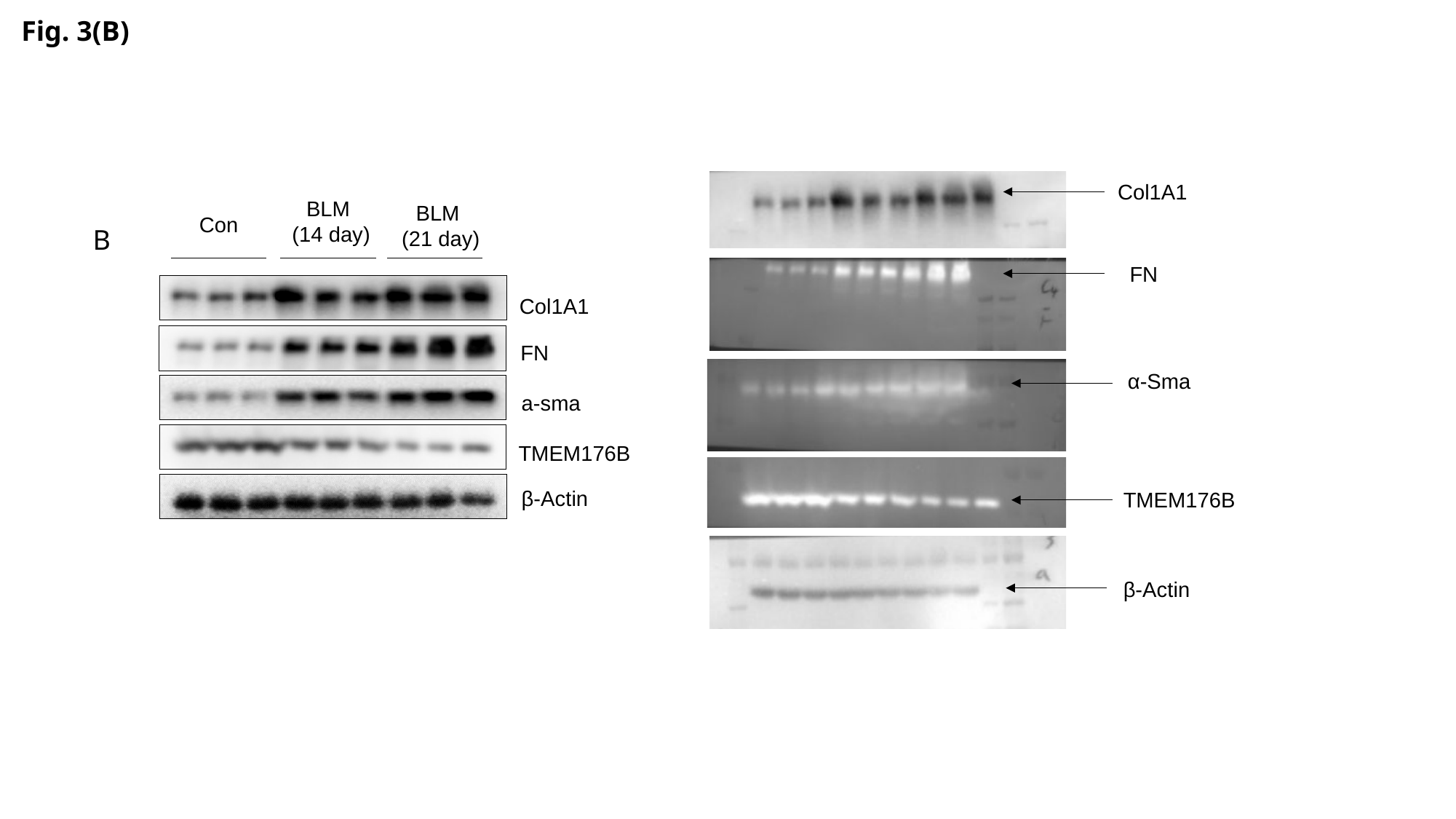

Fig. 3(B)
Col1A1
BLM
 (14 day)
BLM
(21 day)
Con
Col1A1
FN
a-sma
TMEM176B
β-Actin
B
FN
α-Sma
TMEM176B
β-Actin

## Slide 5
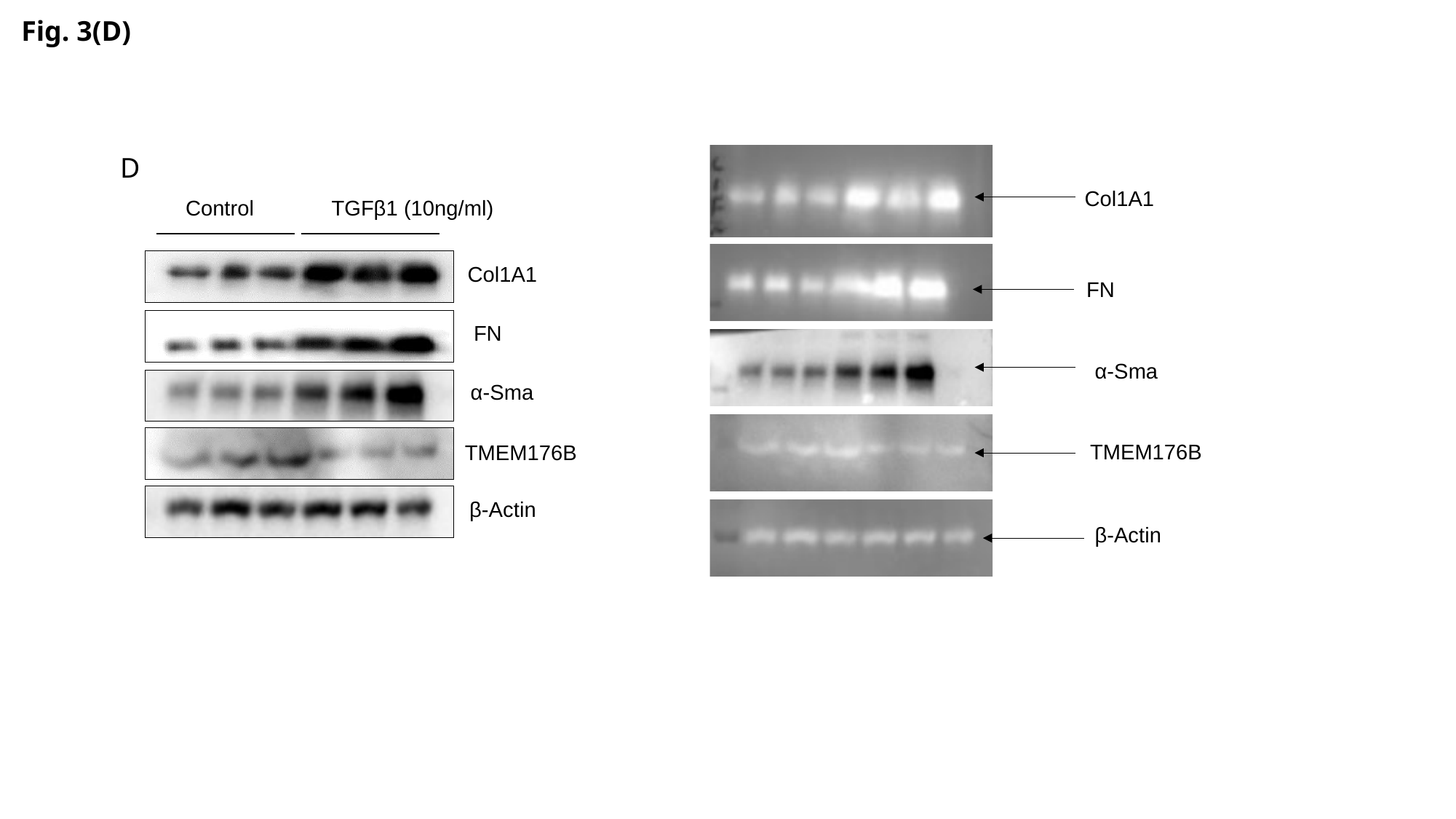

Fig. 3(D)
D
Col1A1
Control
TGFβ1 (10ng/ml)
Col1A1
FN
α-Sma
TMEM176B
β-Actin
FN
α-Sma
TMEM176B
β-Actin

## Slide 6
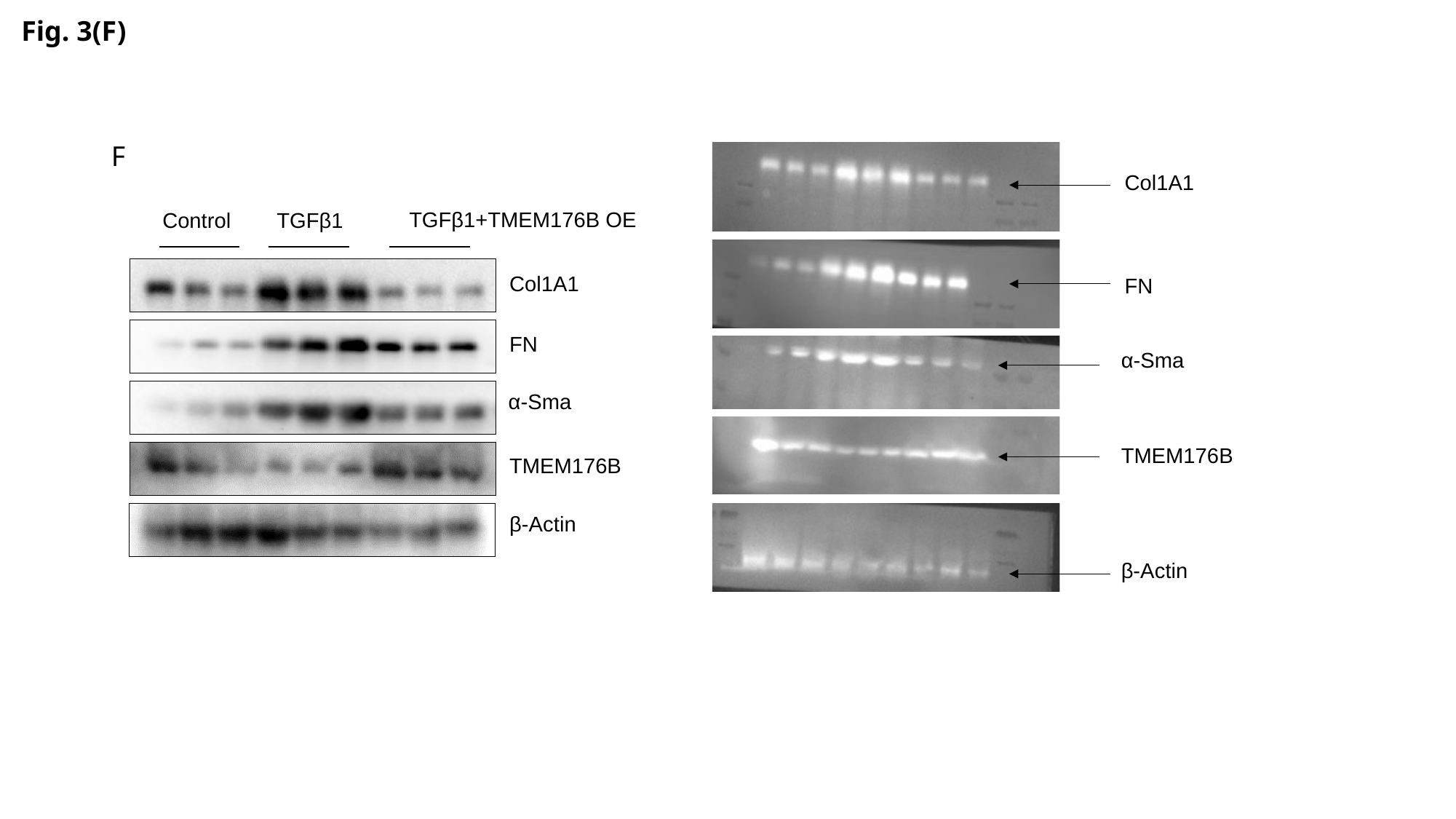

Fig. 3(F)
F
Col1A1
TGFβ1+TMEM176B OE
Control
TGFβ1
Col1A1
FN
α-Sma
TMEM176B
β-Actin
FN
α-Sma
TMEM176B
β-Actin

## Slide 7
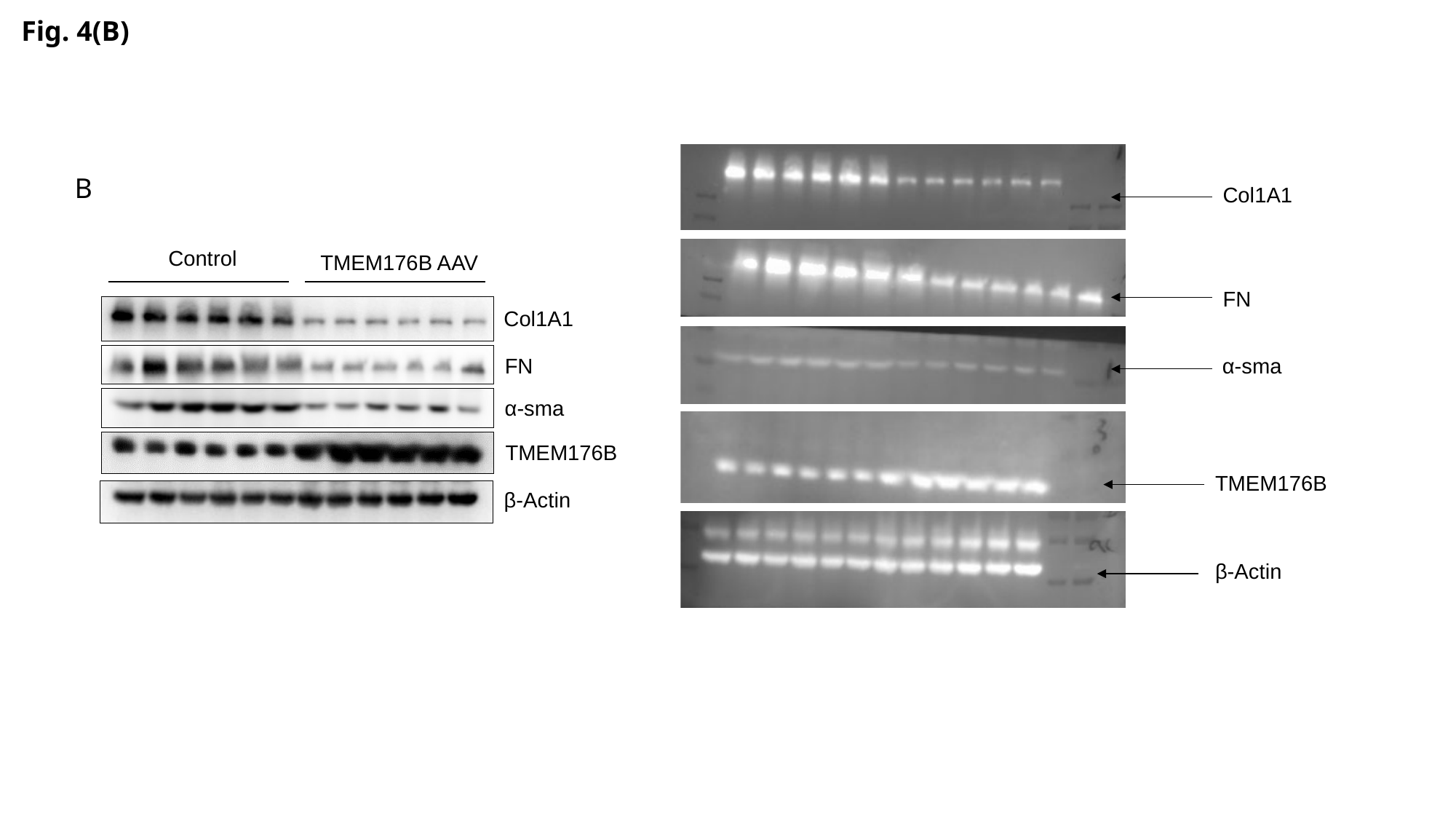

Fig. 4(B)
B
Col1A1
Control
TMEM176B AAV
Col1A1
FN
α-sma
TMEM176B
β-Actin
FN
α-sma
TMEM176B
β-Actin

## Slide 8
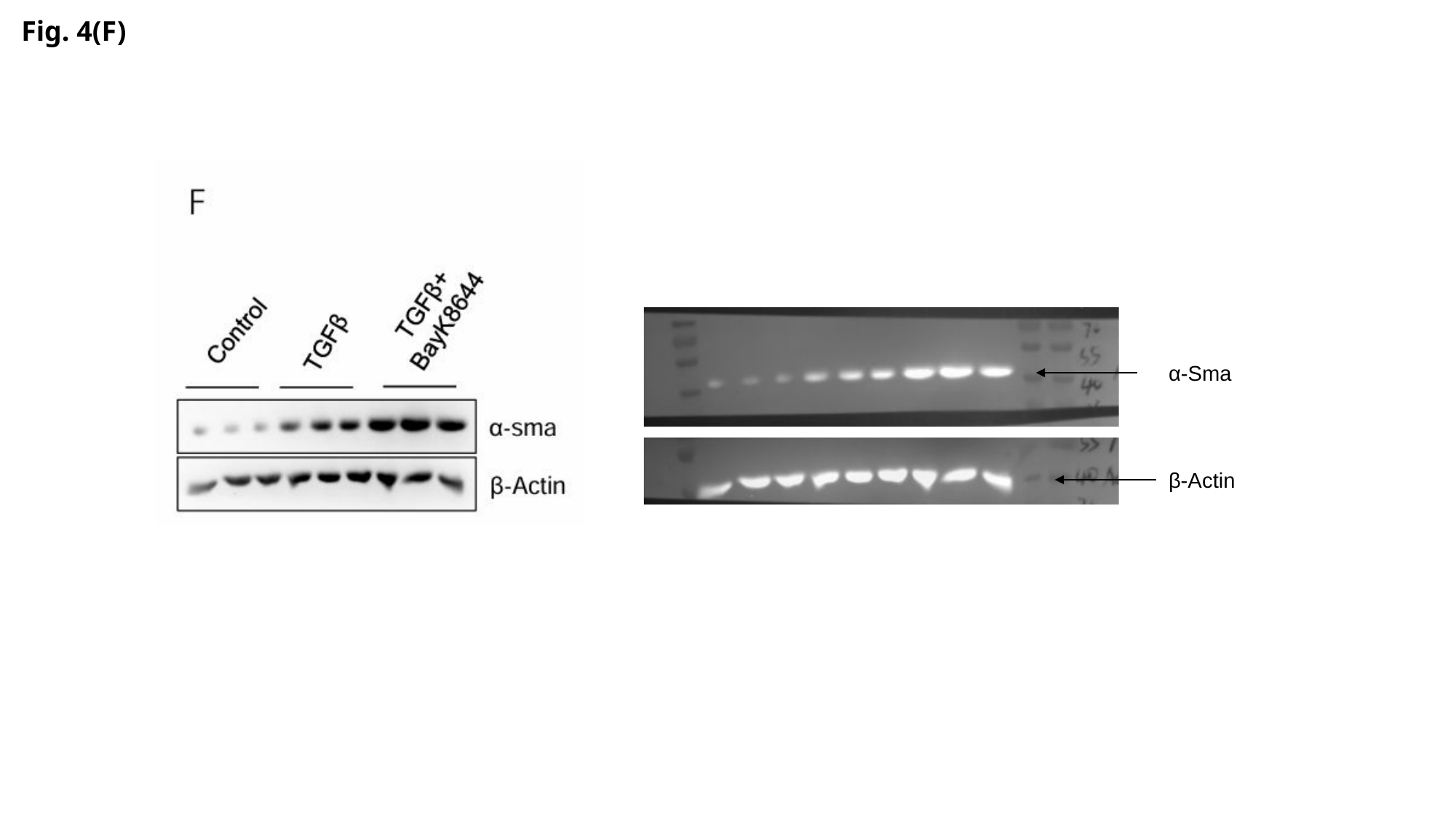

Fig. 4(F)
α-Sma
β-Actin

## Slide 9
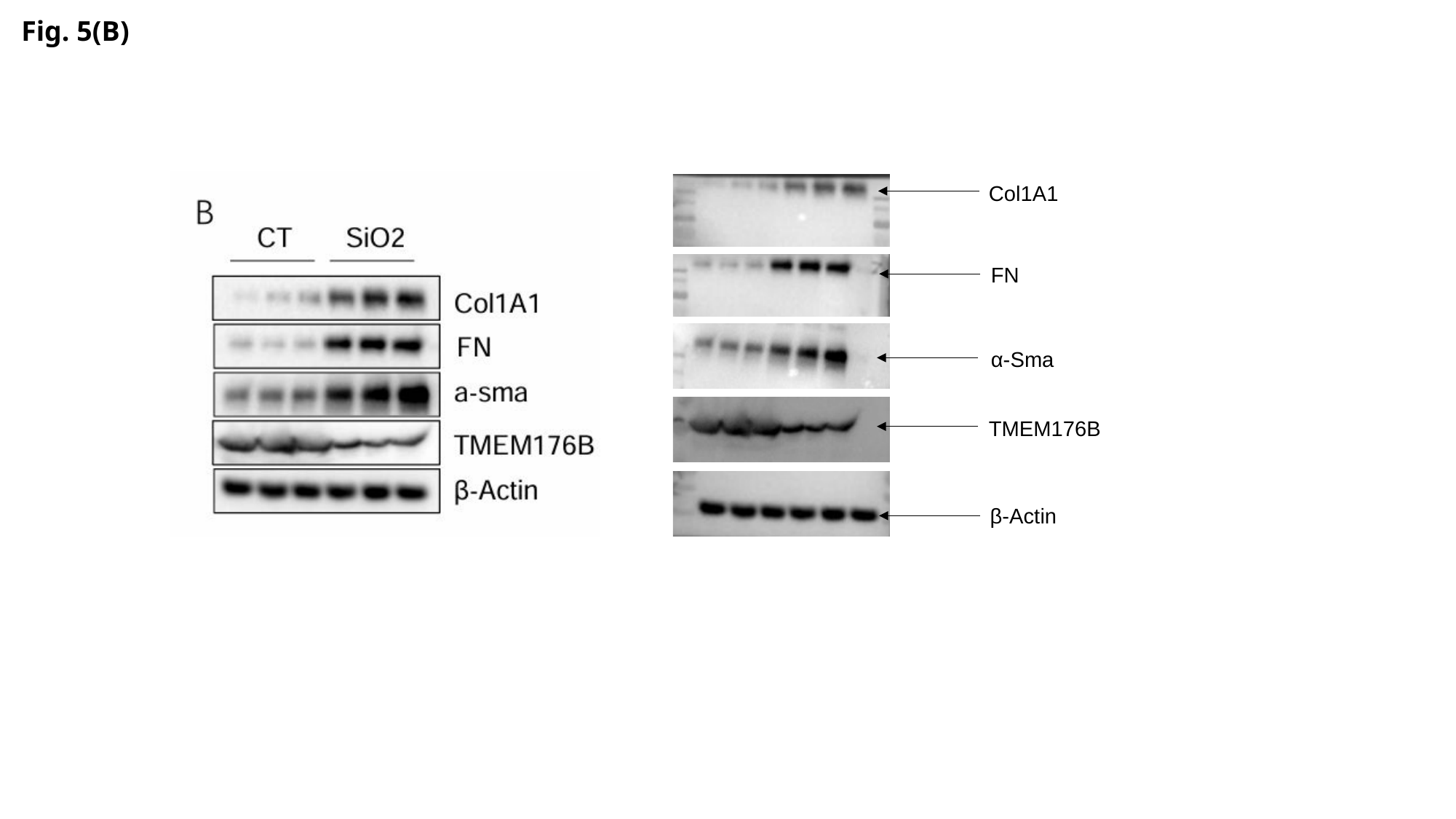

Fig. 5(B)
Col1A1
FN
α-Sma
TMEM176B
β-Actin

## Slide 10
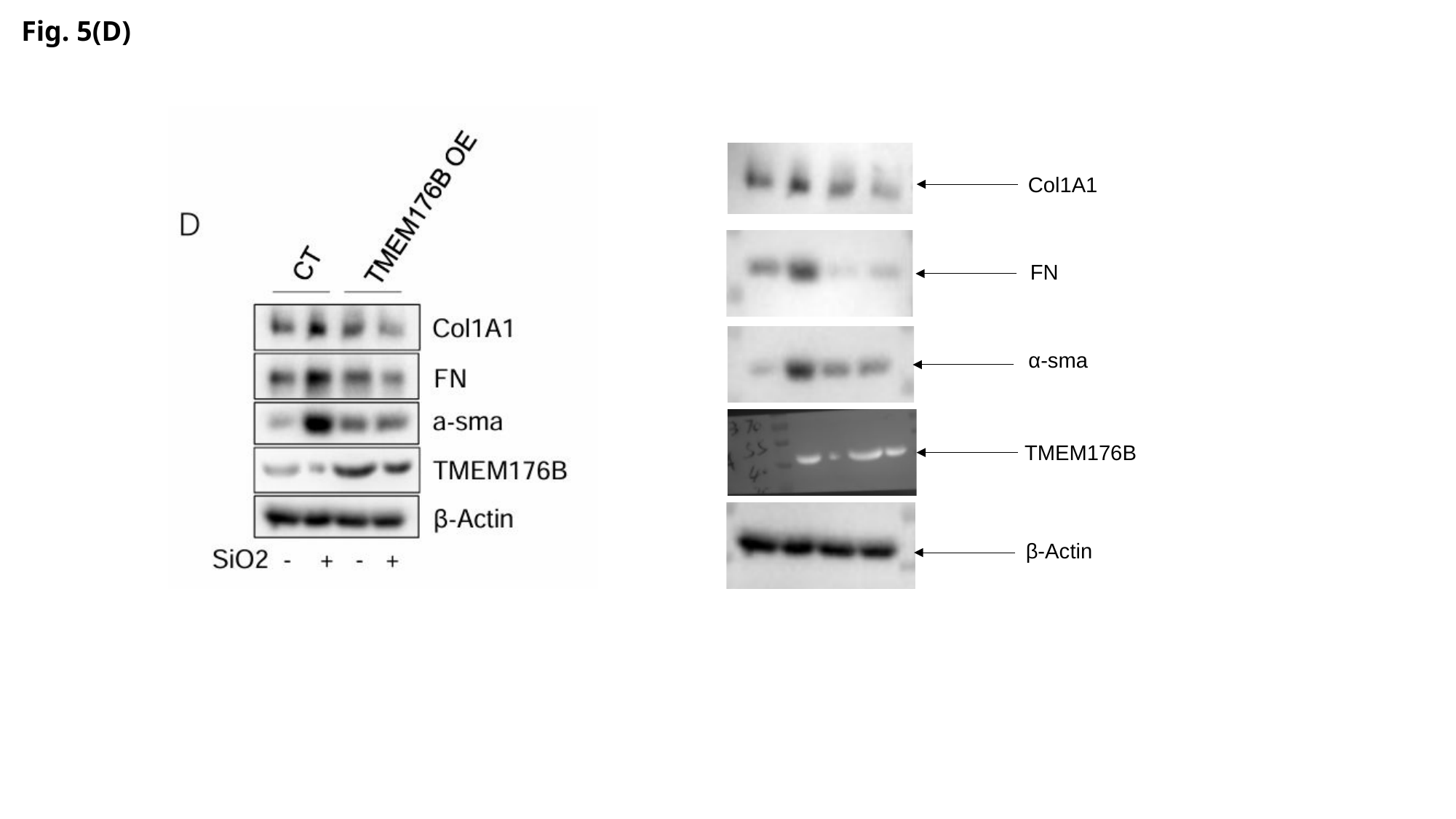

Fig. 5(D)
Col1A1
FN
α-sma
TMEM176B
β-Actin

## Slide 11
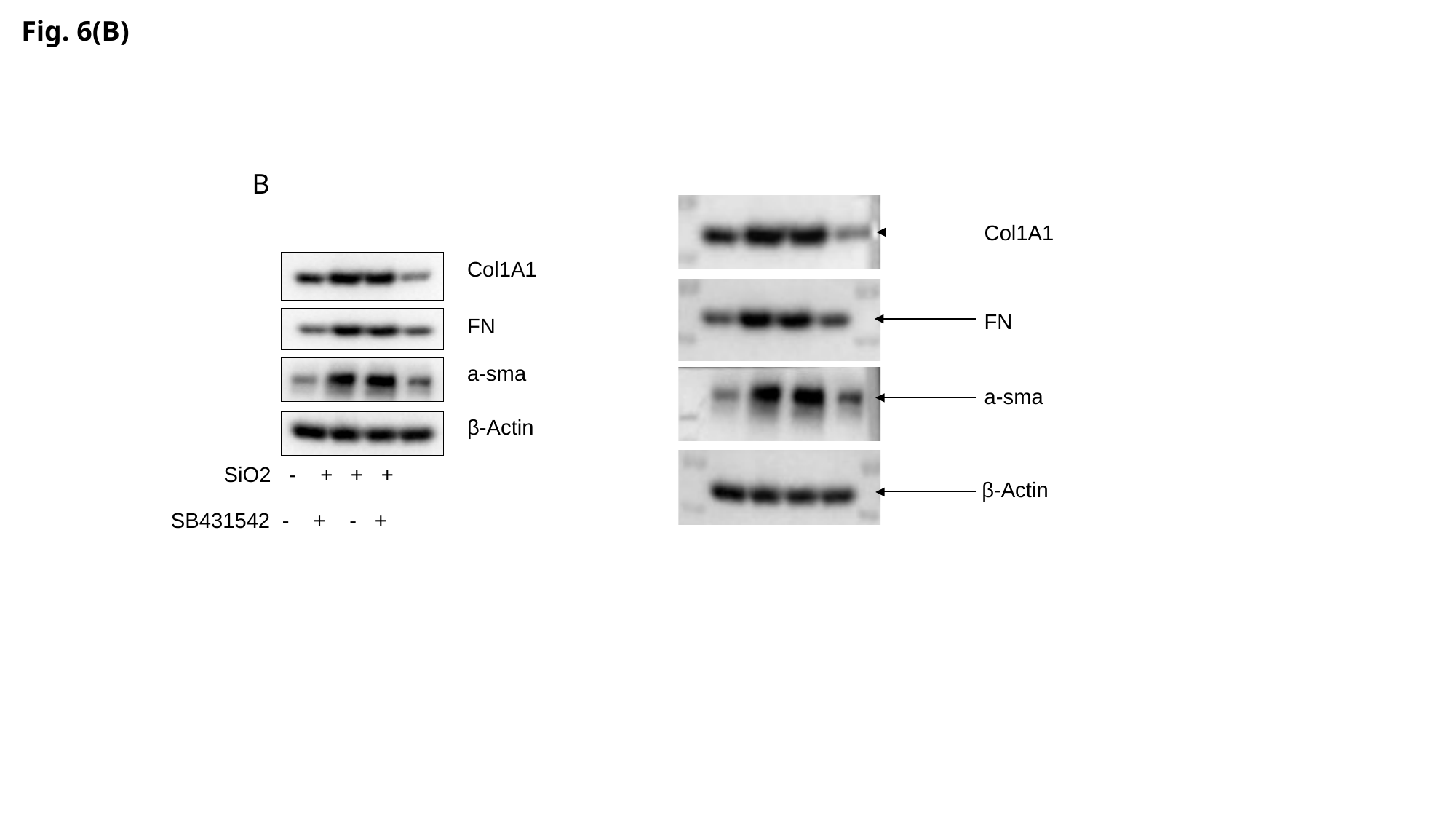

Fig. 6(B)
B
Col1A1
FN
a-sma
β-Actin
SB431542 - + - +
SiO2 - + + +
Col1A1
FN
a-sma
β-Actin

## Slide 12
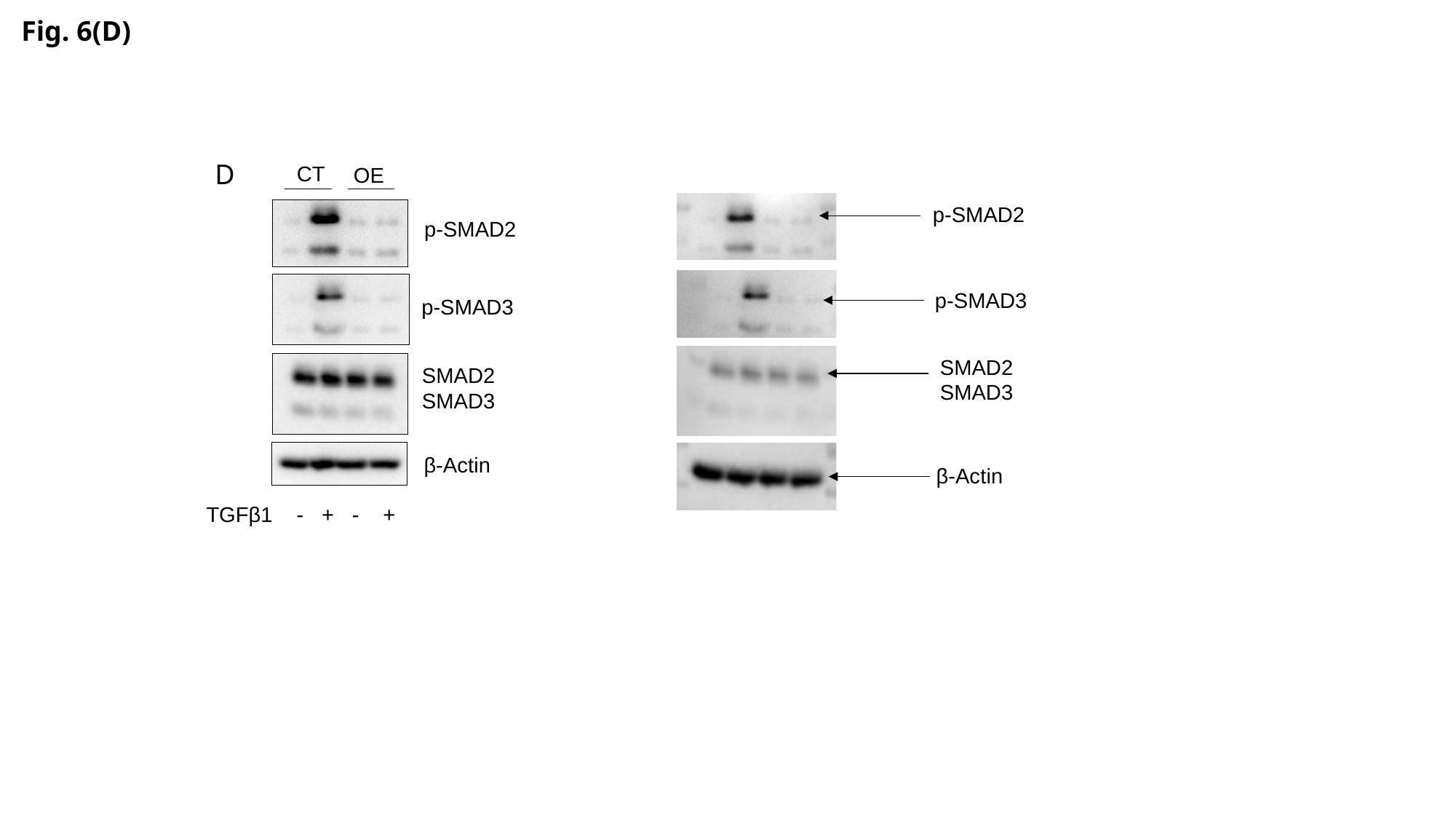

Fig. 6(D)
D
CT
OE
p-SMAD2
p-SMAD3
SMAD2
SMAD3
β-Actin
p-SMAD2
p-SMAD3
SMAD2
SMAD3
β-Actin
TGFβ1 - + - +
